# Supplementary material for: Towards environmental detection, quantification, and molecular characterization of Anopheles stephensi and Aedes aegypti from experimental larval breeding sites
Source: Sci Rep. 2023 Feb 15;13:2729. doi: 10.1038/s41598-023-29657-y (PMC9932160; doi:10.1038/s41598-023-29657-y)
Supplement: Supplementary file 2 — Supplementary Information 2. [file 41598_2023_29657_MOESM2_ESM.docx]

# Supplementary Information for “Towards environmental detection, quantification, and molecular characterization of *Anopheles stephensi* and *Aedes aegypti* from experimental larval breeding sites”

**Supplementary Figure 1.** Standard curves across a tenfold dilution series of gDNA for individual detection of *An. stephensi* (A) or *Ae. aegypti* (B) and simultaneous detection of *An. stephensi* and *Ae. aegypti* (C), across a tenfold gDNA dilution series. Points drawn with black circles are the middle two quartiles of standards with ≥50% detection and are included in the linear regression calculations. Points drawn with blue pluses (+) are outside the middle two quartiles or for standards with <50% detection and are not included in the linear regression calculations.


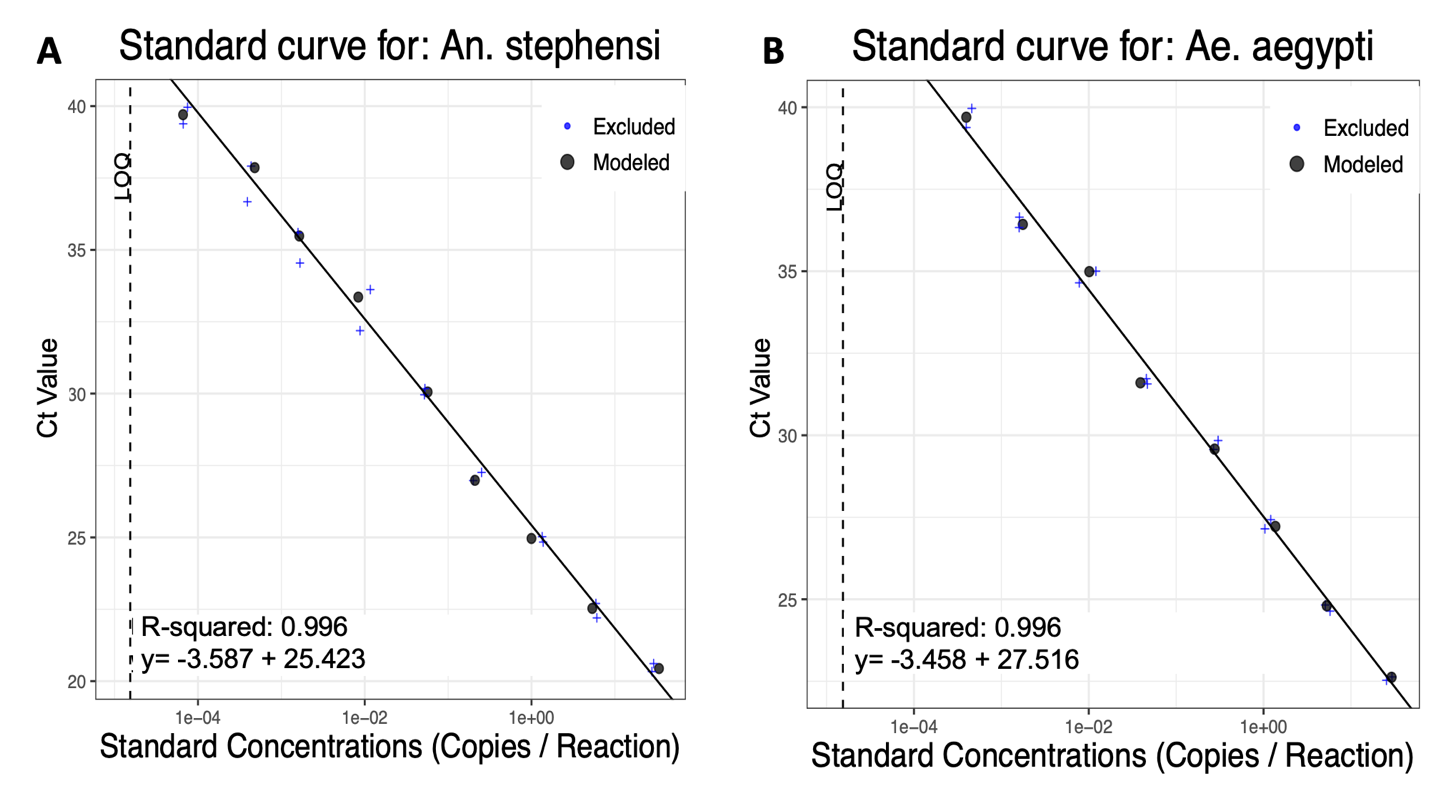


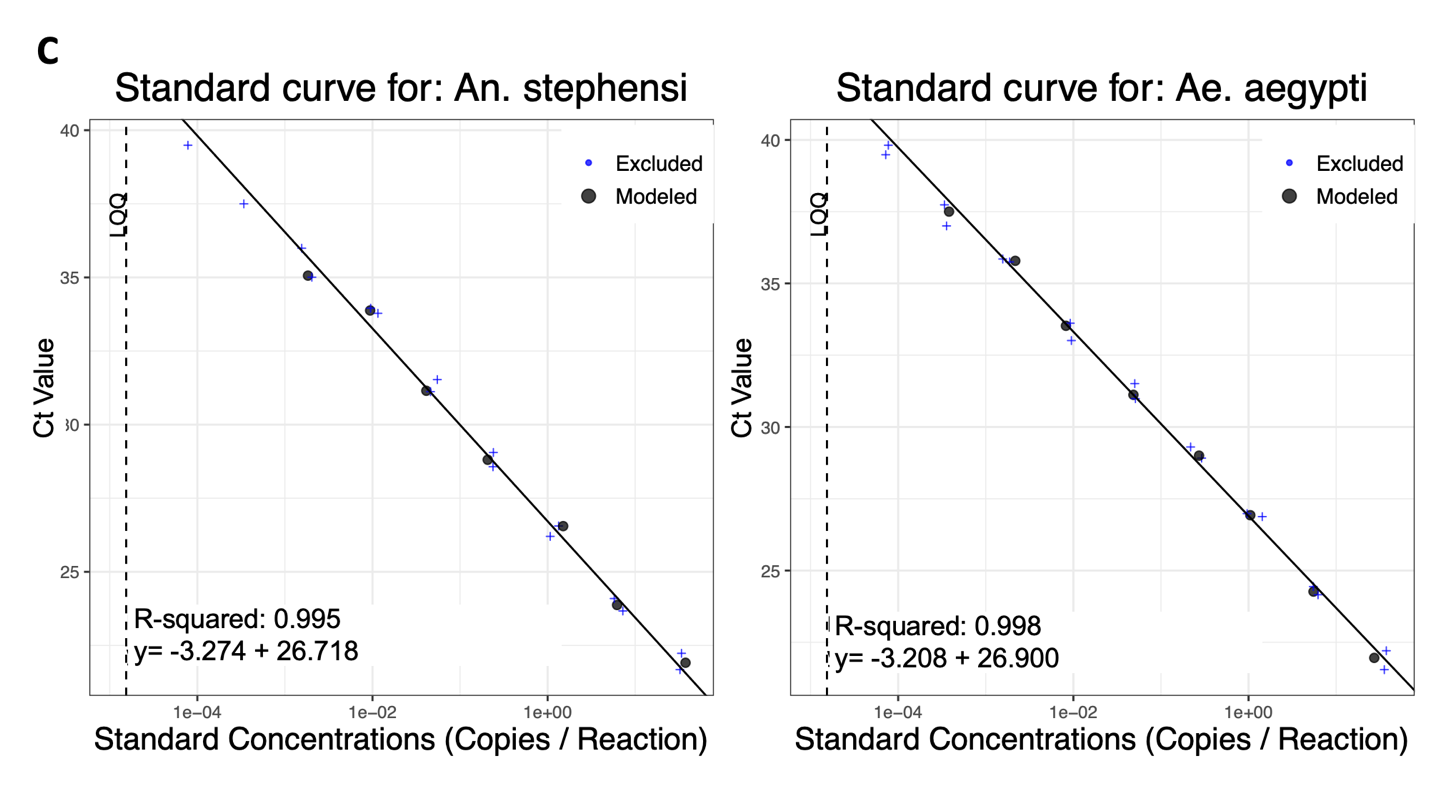


**Supplementary File 1.** qPCR data for 1) TaqMan standard curves; 2) Primer and probe specificities; 3) Experiment 1: 50ml; 4) Experiment 2: 1L; and 5) Experiment 3: eDNA degradation.
